# Analysis of the chemotherapy effect of albumin-bound paclitaxel combined with pirarubicin and cyclophosphamide on breast cancer and the effects of PCR, ORR, and CBR

**DOI:** 10.12669/pjms.41.3.9861

**Published:** 2025-03

**Authors:** Youzhong Liu, Ke Gong, Songlin Yuan, Yong Xu

**Affiliations:** 1 Youzhong Liu, Department of Thyroid and Breast Surgery, Changde Hospital, Xiangya School of Medicine, Central South University (The First People’s Hospital of Changde City), Changde 415000, Hunan, China; 2 Ke Gong, Department of Thyroid and Breast Surgery, Changde Hospital, Xiangya School of Medicine, Central South University (The First People’s Hospital of Changde City), Changde 415000, Hunan, China; 3 Songlin Yuan, Department of Thyroid and Breast Surgery, Changde Hospital, Xiangya School of Medicine, Central South University (The First People’s Hospital of Changde City), Changde 415000, Hunan, China; 4 Yong Xu, Department of Thyroid and Breast Surgery, Changde Hospital, Xiangya School of Medicine, Central South University (The First People’s Hospital of Changde City), Changde 415000, Hunan, China

**Keywords:** Albumin-bound paclitaxel, Breast cancer, Cyclophosphamide, Neoadjuvant chemotherapy, Pirarubicin

## Abstract

**Objective:**

To explore the chemotherapy effect of albumin-bound paclitaxel combined with pirarubicin and cyclophosphamide on breast cancer and the effects of PCR, ORR and CBR.

**Methods:**

This was a retrospective study. During the period from January 2022 to December 2023, ninety patients with breast cancer who were treated in The First People’s Hospital of Changde City were included as the research objects. Based on the principle of randomized control, the above patients were divided into the control group (45 cases, treated with pirarubicin and cyclophosphamide) and the observation group (45 cases, treated with albumin-bound paclitaxel combined with pirarubicin and cyclophosphamide) by the random number table. Compared the chemotherapy effect and safety of the two groups.

**Results:**

After treatment, the PCR, ORR and CBR of the observation group were higher than those of the control group, while the levels of CA152, CA125, TPS, HER-2, Ki-67 and EGFR were lower than those of the control group, and the differences were statistically significant (*P*<0.05). The incidence of adverse reactions was 33.33% (15/45), which was slightly higher than that of 28.89% (13/45) in the control group, the difference was not statistically significant (*P*>0.05).

**Conclusion:**

Albumin-bound paclitaxel combined with pirarubicin and cyclophosphamide is a safe regimen that can further enhance the effect of neoadjuvant chemotherapy in the treatment of breast cancer, and it has a certain value for dissemination in the treatment of breast cancer can further enhance the effect of neoadjuvant chemotherapy in patients, which is of certain value for clinical promotion.

## INTRODUCTION

Breast cancer is the most frequent malignancy in women and is primarily attributed to the uncontrolled proliferation of breast epithelial cells. Data from relevant studies^1^ show that the global incidence of breast cancer is as high as 24.2%, of which more than 50% occurs in developing countries. Breast cancer ranks first in the incidence rate of all female malignant tumors, presently showing a rising trend year by year in China.^2^ The majority of patients in the early stage of breast cancer are accompanied by varying degrees of breast lumps, nipple discharge and axillary lymph node enlargement; they also develop distal metastasis of tumor cells in their body and then involve multiple organs with the progression of the disease, posing a severe threat to their lives.

However, a study^3^ pointed out that despite the current high incidence rate, breast cancer is one of the solid tumors with the optimal clinical efficacy at present as medical treatment continues to develop. Surgery is currently considered to be the preferred modality for breast cancer patients, with most of them generally having a better prognosis after radical mastectomy.^4^ However, another study^5^ reveals a tendency for breast cancer to ravage increasingly younger women in recent years. Most of the non-menopausal patients expect post-treatment fertility preservation. For this reason, conventional radical mastectomy may be an unlikely option to address the clinical needs of patients. Of the numerous surgical modalities currently available in conjunction with breast-conserving treatment, neoadjuvant chemotherapy is a key one.

Aside from fully adapting to the patients’ demand for breast preservation, it is of positive significance for the treatment of some patients with large tumor diameters and axillary lymph node metastases.^6^ Anthracyclines, paclitaxel and cyclophosphamide are commonly used in neoadjuvant chemotherapy for breast cancer at the present stage. They have been confirmed by relevant studies for their efficacy when used alone in neoadjuvant chemotherapy for breast cancer.^7^ Yet, no in-depth study has been conducted on the efficacy and safety of the combined application of the three. We gave priority in this study to investigate the chemotherapy effect of albumin-bound paclitaxel combined with pirarubicin and cyclophosphamide on breast cancer and the effects of PCR, ORR and CBR, aiming to further optimize neoadjuvant chemotherapy regimen for breast cancer patients and improve their prognosis.

## METHODS

This was a retrospective study. Ninety cases of breast cancer patients admitted to The First People’s Hospital of Changde City from January 2022 to December 2023, and divided into the control group (n=45) and the observation group (n=45) according to different treatment methods.

### Ethical Approval:

The study was approved by the Institutional Ethics Committee of The First People’s Hospital of Changde City (No.:2022-257-01; date: November 21, 2022), and written informed consent was obtained from all participants.

The diagnostic criteria were as follows: referring to the Guidelines of CSCO on Diagnosis and Treatment of Breast Cancer^8^, i.e.,

- presence of primary tumor confirmed by bilateral mammogram, ultrasound, CT, MRI and hollow needle puncture;
- visible regional lymph node metastasis and distant lesions on physical examination;
- conformity to the pathologic diagnostic criteria of breast cancer.

### Inclusion criteria:

- Patients who met the diagnostic points of the above guidelines.
- Those with indications related to neoadjuvant chemotherapy.^9^
- Those with an expected life cycle greater than three months by clinical assessment.
- Those who had not received other neoadjuvant chemotherapy drugs within one month prior to enrollment in this study.

### Exclusion criteria:

- Patients with benign breast tumors.
- Those with cystic hyperplasia or mastitis of the breast.
- Those with clinical stages of grade I to II.
- Those who voluntarily undergo radical mastectomy.
- Patients with acute illness, lactic acidosis, connective tissue disorders.

Patients in the control group ranged in age from 40 to 60 years, with an average age of 50.25±2.15 years, and tumor diameter ranged from 5 to 10 cm, with an average age of 7.52±0.38 cm. In terms of menstruation, there were 30 cases of menopause and 15 cases of premenopause; and in terms of clinical stage of the tumor, there were 9 cases of IIIA stage, 17 cases of IIIB stage, 10 cases of IIIC stage and 9 cases of IV stage. Those in the observation group ranged in age from 41 to 58 years, with an average age of 49.74±2.86 years, and tumor diameter ranged from 6 to 9cm9 cm, with an average age of 7.13±0.17 cm. In terms of menstruation, there were 31 cases of menopause and 14 cases of premenopause; and in terms of clinical stage of the tumor, there were 10 cases of IIIA stage, 20 cases of IIIB stage, 8 cases of IIIC stage and 7 cases of IV stage. No statistically significant difference was found in terms of baseline data between the two groups (*P*>0.05), which were comparable.

### Methods:

A combined treatment of pirarubicin and cyclophosphamide was used. Pirarubicin (manufacturer: Shenzhen Main Luck Pharmaceuticals Inc.; NMPA approval No.: H10930105; strength: 50 mg) was injected intravenously on the first day at 90 mg/m^2^/dose, with 21 d for a course of treatment; cyclophosphamide (manufacturer: Baxter Oncology GmbH (Germany), NMPA approval No.: HJ20160467; strength: 2 g) was injected intravenously on the first day at 600 mg/m^2^/dose, with 21 days for a course of treatment. The efficacy was assessed after four courses of continuous medication.

Albumin-bound paclitaxel combined with pirarubicin and cyclophosphamide was used for treatment, of which the usage and dosage of pirarubicin and cyclophosphamide were the same as that of the control group. Albumin-bound paclitaxel (manufacturer: Zhejiang Hisun Pharmaceutical Co., Ltd.; NMPA approval No.: H20213539; strength: 100 mg) was injected intravenously at 100–150 mg/m^2^/dose on the 1st, 8th, and 15th day of chemotherapy, with every 28 d was a course of treatment. The efficacy was assessed after 4 courses of continuous medication.

### Observation indexes:

Primary assessment indexes: The pathological complete response (PCR) rate, objective response rate (ORR), and clinical benefit rate (CBR) of the two groups were calculated using the improvement of solid tumors after treatment as the main efficacy assessment criteria. Breast ultrasound revealed that PCR was determined when no histological evidence of malignancy was found in the primary lesion of breast cancer after treatment, or only carcinoma in situ was present, and there was no residual infiltrating tumor cell on the pathological examination of surgical specimens from axillary lymph nodes.^10^ Referring to the Response Evaluation Criteria in Solid Tumors (RECIST1.1),^11^ complete response (CR) was determined when the target lesion disappeared, no new lesions were found, and the level of various tumor markers remained in the normal range for more than four weeks. Partial response (PR) was determined if the target lesion diameter and reduction were greater than 30% and the level of tumor markers remained in the normal range for more than four weeks; And stable disease (SD) was judged if the target lesion diameter was reduced by 20–30%. ORR=(CR+PR)/total number of cases×100%, DCR=(CR+PR+SD)/total number of cases×100%, and CBR was determined when DCR was maintained for more than six months.

### Secondary assessment indexes:

Tumor markers, immunohistochemical factor levels and the incidence of adverse reactions before and after treatment were used as secondary evaluation indexes for the efficacy of neoadjuvant chemotherapy in the two groups. For the first two indexes, peripheral venous blood of patients was collected as test samples, which were uniformly anticoagulated by EDTA and then centrifuged at a speed of 1000 r/ minute and a radius of 0.5 cm for one minute for testing. Tumor markers included three items of glycan antigen 153 (CA152), CA125, and tissue polypeptide specific antigen (TPS), and immunohistochemical factors included three items of human epidermal growth factor receptor-2 (HER-2), cell proliferation-related protein (Ki-67), and epidermal growth factor receptor (EGFR). In this study, the adverse reactions of the two groups after treatment mainly included such five kinds as hematologic disorder, gastrointestinal reaction, cardiotoxicity, hepatic insufficiency, and myelosuppression. The sum percentage of the above adverse reactions was taken as the final incidence rate for comparison.

### Statistical analysis:

All data were statistically analyzed using the SPSS 26.0 software (SPSS Inc., Chicago, IL, USA). Measurement data were expressed as mean±standard deviation (*X̅*±*S*), and an independent sample *t* test was used for comparison between groups. Enumeration data were expressed as the number of cases (n) and rate (%), and the chi-squared χ^2^ test was used for comparisons between groups. A *P*-value <0.05 indicated a statistically significant difference.

## RESULTS

After treatment, the PCR, ORR and CBR of the observation group were higher than those of the control group, with statistically significant differences (*P*<0.05), Table-I. No statistically significant difference was found in the levels of tumor markers between the two groups before treatment. After treatment, the indexes of the two groups were lower than before treatment, and the levels of CA152, CA125 and TPS were lower than those of the control group, with statistically significant differences (*P*<0.05), Table-II.

**Table-I:** Comparison of chemotherapy effect between the two groups (n, %).

Group	n	PCR	ORR	CBR
Observation group	45	28 (62.22)	30 (66.67)	20 (44.44)
Control group	45	21 (46.67)	23 (51.11)	12 (26.67)
*x^2^*	-	4.875	5.000	6.891
*P*	-	0.027	0.025	0.009

**Table-II:** Comparison of tumor marker levels between the two groups (*χ̅±S*).

Group	n	CA152 (U/ml)		CA125 (U/ml)		TPS (mU/ml)	

		Before treatment	After treatment	Before treatment	After treatment	Before treatment	After treatment
Observation group	45	50.32±2.28	20.12±1.15^a^	70.16±2.27	39.45±1.14^a^	82.74±2.38	52.11±1.14^a^
Control group	45	50.11±2.46	26.11±1.71^a^	70.46±2.33	44.36±1.28^a^	82.16±2.46	57.45±1.39^a^
*t*	-	0.420	19.499	0.619	19.216	1.137	19.927
*P*	-	0.676	0.000	0.538	0.000	0.259	0.000

***Note:***

a *P*<0.05 under comparisons of this group before and after treatment.

No statistically significant difference was found in the levels of immunohistochemical factors between the two groups before treatment. After treatment, the indexes of the two groups were lower than before treatment, and the levels of HER-2, Ki-67 and EGFR were lower than those of the control group, with statistically significant differences (*P*<0.05), Table-III. After treatment, the incidence of adverse reactions was 33.33% (15/45), which was slightly higher than that of 28.89% (13/45) in the control group, with a statistically significant difference (*P*>0.05), Table-IV.

**Table-III:** Comparison of immunohistochemical factors between the two groups (*χ̅±S*).

Group	n	HER-2 (ug/L)		Ki-67 (%)		EGFR (ug/L)	

		Before treatment	After treatment	Before treatment	After treatment	Before treatment	After treatment
Observation group	45	15.25±5.13	5.16±1.14^a^	16.44±1.16	9.25±0.33^a^	16.44±2.31	10.22±1.17^a^
Control group	45	15.33±5.25	8.23±1.36^a^	16.32±1.38	12.31±0.27^a^	16.35±2.45	13.36±1.28^a^
*t*	-	0.073	11.605	0.447	48.143	0.179	12.146
*P*	-	0.942	0.000	0.656	0.000	0.858	0.000

***Note:***

a *P*<0.05 under comparisons of this group before and after treatment.

**Table-IV:** Comparison of the incidence of adverse reactions between the two groups (n, %).

Group	n	Hematologic disorder	Gastrointestinal reaction	Cardiotoxicity	Hepatic insufficiency	Myelosuppression	Overall incidence
Observation group	45	4 (8.89)	5 (11.11)	1 (2.22)	2 (4.44)	3 (6.67)	15 (33.33)
Control group	45	3 (6.67)	3 (6.67)	2 (4.44)	2 (4.44)	3 (6.67)	13 (28.89)
*x^2^*	-	-	-	-	-	-	0.460
*P*	-	-	-	-	-	-	0.498

## DISCUSSION

The present study revealed that both groups achieved certain therapeutic effects after varying modes of chemotherapy, and their tumor foci were evidently improved compared with those before treatment by breast ultrasound. The reason may be that pirarubicin acts directly on the cell nucleus, resulting in a pharmacological mechanism that interferes with the DNA transcription process of tumor cells, hinders the formation of mRNA, and inhibits DNA and RNA synthesis. As a cell cycle non-specific drug, pirarubicin ensures the efficacy of a variety of transplantable tumors while reducing cardiotoxicity to a certain extent.^12^ This study showed that the observation group had a better effect after being treated with the combination of three drugs, and its corresponding PCR, ORR, and CBR were higher than those of the control group, which was considered to be related to the pharmacological mechanism of albumin-bound paclitaxel. Paclitaxel analogs are currently popular drugs for the treatment of advanced metastatic breast cancer. They mainly act on tumor microtubules and achieve dynamic reorganization of the microtubule network by inhibiting interphase and mitotic cell function, thus promoting tumor cell division. A multicenter clinical study^13^ noted a higher PCR and greater clinical benefit in patients treated with albumin-bound paclitaxel compared to first-line chemotherapy with docetaxel, which is generally consistent with the findings of this study.

Presently, the prognosis of breast cancer patients is considered to have a close bearing on the development of their disease. Data from relevant studies^14^ reveal a five years survival rate of up to 98% for patients with in situ breast cancer and 86% for early invasive cancer, but less than 28% of the five years survival rate for those who have developed distal tumor metastasis. At the present stage, anthracyclines and paclitaxel are commonly used in neoadjuvant chemotherapy for breast cancer, the mechanism of which is primarily to change and inhibit the biochemical metabolism of cancer cells, thus inhibiting the overproliferation of cells. Cyclophosphamide is a broad-spectrum antitumor agent that can be hydrolyzed by phosphoramidases and phosphatases present in tumors and activated to phosphoramidite nitrogen mustard, which the effect of anti-DNA and interfering with the function of RNA by cross-linking with DNA.^15^ It has been established^16^ that pirarubicin resembles cyclophosphamide in its pharmacological mechanism, and that their combination may further enhance the efficacy of chemotherapy in patients. Yet, another study^17^ pointed out that the risk of adverse reactions is higher when the two are combined, with sarcopenia as a risk factor affecting their efficacy. Ordinary paclitaxel is insoluble in water, and co-solvents are generally required.

In contrast, albumin-bound paclitaxel is a particle with a diameter of only 130nm, which eliminates the need for antiallergic pretreatment before injection; and its pharmacological components can be highly targeted to tumor foci in order to enhance efficacy while reducing the adverse effects associated with co-solvents.^18^ As pointed out by the National Comprehensive Cancer Network (NNCN) in recent years, albumin-bound paclitaxel, characterized by high efficiency, hypoallergenicity, and safety, can be used as a novel type of drug currently used in neoadjuvant chemotherapy for a variety of tumors.^19^

Our study revealed that the tumor markers and immunohistochemical factors in the observation group were closer to the normal level after the three-drug combination treatment, and the corresponding CA152, CA125, TPS, HER-2, Ki-67 and EGFR were lower than those in the control group, evidencing the better efficacy of the three-drug combination than the two-drug combination. Myelosuppression is the predominant adverse effect of albumin-bound paclitaxel. And related studies^20^ pointed out that leukopenia and neutropenia are the primary types of adverse reactions of the drug. In this study, the incidence of adverse reactions in the two groups was compared to further verify the safety of the three-drug combination. The results showed that there was no significant difference between the incidence rates of the two groups, proving the high safety of the three-drug combination.

### Limitations:

However, the results of this study are limited by the fact that the severity of adverse reactions was not graded. In this regard, the grading of adverse reactions in patients between the three-drug combination and conventional neoadjuvant chemotherapy regimens can be further demonstrated in subsequent clinical studies based on the present study, thus further demonstrating the safety of the dosing regimen.

## CONCLUSIONS

In conclusion, albumin-bound paclitaxel combined with pirarubicin and cyclophosphamide is a safe regimen that can further enhance the effect of neoadjuvant chemotherapy in the treatment of breast cancer, and it has a certain value for dissemination in the treatment of breast cancer can further enhance the effect of neoadjuvant chemotherapy in patients, which is of certain value for clinical promotion.

### Authors’ Contributions:

**YL** and **KG:** Carried out the studies, participated in collecting data, and drafted the manuscript, and are responsible and accountable for the accuracy or integrity of the work. **SY** and **YX:** Performed the statistical analysis and participated in its design. Critical review. All authors read and approved the final manuscript.
